# Complications of primary total hip arthroplasty among patients with rheumatoid arthritis, psoriatic arthritis, ankylosing spondylitis, and primary osteoarthritis

**DOI:** 10.1186/s12891-022-05891-9

**Published:** 2022-10-19

**Authors:** Qiang Lian, Yun Lian, Kangxian Li, Qinfeng Yang, Kunlian Li, Yiqiao Zheng, Haibing Liu, Zhanjun Shi, Jian Wang

**Affiliations:** 1 Division of Orthopaedic Surgery, Department of Orthopaedics, Nanfang Hospital, Southern Medical University, 1838 Guangzhou Avenue, Guangzhou, 510515 Guangdong China; 2 First Affiliation Hospital of Nanchang University, Nanchang, China; 3 Goodwill Hessian Health Technology Co., Ltd, Beijing, 100007 China; 4 Department of Orthopaedic, Affiliated Hengyang Hospital, Southern Medical University (Hengyang Central Hospital), Hengyang, 421001 China

**Keywords:** Inflammatory arthropathies, Hip replacement, Primary osteoarthritis, Nationwide inpatient sample

## Abstract

**Background:**

Limited information exists comparing the perioperative complications of the different inflammatory arthropathies (IAs) after total hip arthroplasty (THA). Our study was aimed to (1) compare perioperative complications and (2) determine the most common complications between the different IA subtypes compared with patients with primary osteoarthritis (OA) undergoing primary THA and (3) find whether the difference in postoperative complications also exists between different IA after THA.

**Methods:**

The Nationwide Inpatient Sample (NIS) was used to identify patients with Rheumatoid arthritis (RA), psoriatic arthritis, ankylosing spondylitis (AS), and primary OA undergoing unilateral THA between 2005 and 2014. Preoperative diagnosis, comorbidities, and postoperative complications were determined using the International Classification of Disease Clinical Modification version 9 codes. The prevalence of perioperative complications was compared between patients with IA and primary OA and between patients with different IA.

**Results:**

When compared with patients with primary OA, patients with RA had significantly more postoperative surgical and medical complications. Yet there are just several medical complications differences exist between PA and primary OA or AS and primary OA, including stroke and acute renal failure for psoriatic arthritis and urinary tract infection and pneumonia for AS. What is more, there were also several differences in perioperative medical complications seen in patients with different IA.

**Conclusion:**

Except for patients with RA, the differences in perioperative complications was small between patients with IA and primary OA and between patients with different types of IA.

**Supplementary Information:**

The online version contains supplementary material available at 10.1186/s12891-022-05891-9.

## Background

One of the etiologies underlying most primary total hip arthroplasty (THA) procedures is primary OA, which proved to be the commonest kind of arthritis of the hip [1, 2]. Therefore, most previous studies regarding prognosis after THA in patients with arthritis are focused on osteoarthritis, leading to the limited available data on outcomes of THA of inflammatory diseases, such as rheumatoid arthritis, and ankylosing spondylitis, and psoriatic arthritis.

Inflammatory arthritis and primary OA of the hip are pathologically distinct diseases with different proper medical management and prognosis, resulting in variable expected outcomes for patients undergoing THA [3–5]. Being the commonest form of inflammatory arthritis in America, rheumatoid arthritis compared with primary OA generally in most of the existing studies on inflammatory arthritis and THA procedures [6–9]. Increased risk of infection have emphasized in several literatures on outcomes of THA; however, rates of postoperative complications and revision has been reported to vary in rheumatoid arthritis [4, 6, 8, 10–14].For example, although previous studies have indicated that the incidence of venous thromboembolism (VTE) after THA in the management of primary OA is 3 to 10 times higher than that in the management of rheumatoid arthritis, other meta-analyses showed no difference in VTE rates [4, 6, 8, 10–14]. In addition, some studies with a large database comparing the revision rates after THA in the management of rheumatoid arthritis and primary OA indicated no significant difference in revision rates, while other meta-analyses demonstrated a slight increase in the odds ratio of 1.24 in 5-year revision rates [4, 6, 8, 10–14]. For patients with other types of inflammatory arthritis, such as ankylosing spondylitis or psoriatic arthritis, data available on the outcomes or complications of THA are insufficient. The prevalence of these IA is low, however, complications, such as pulmonary embolism (PE) and infection after THA can lead to devastating consequences. To study the complication rates of these populations to enable assessment of risk adequately before THA, we compared the outcomes and postoperative complications of primary THA performed for patients with inflammatory arthritis with those for patients with primary OA by employing a national database. What is more, limited studies had discussed whether the difference in postoperative complications also exists between different IA due to the low prevalence of these IA in THA.

We hypothesized that the perioperative complications of IA and primary OA were significantly different and that different IA also had many different perioperative complications.

## Methods

### Data source

The NIS database was used to acquire data to identify the information of patients who underwent THA between 2005 to 2014 according to procedure codes of the International Classification of Diseases, Ninth Revision, Clinical Modification (ICD-9-CM). The NIS database is a large American inpatient care database, including data on millions of hospitalized patients, such as access, diagnoses, operations, outcomes, and charges.

### Data collection

Patients who underwent THA from 2005 to 2014 were identified with ICD-9-CM procedure code 81.51, then divided into different cohorts based on the ICD-9-CM diagnostic codes for RA (code 714.0–714.4, 714.8, 714.9), AS (code 720.0–720.2, 720.8, 720.9), psoriatic arthritis (code 696.0), or primary OA (code 715.1–715.3, 715.8, 715.9, and without a diagnosis of inflammatory arthritis). Basic demographics were queried in each cohort, including sex, age group (≤65admission,66 to 79 years, ≥80 years), admission, and obesity. To grade the comorbidity, Charlson Comorbidity Index (CCI) was calculated in each cohort. Postoperative complications were divided into medical complications and surgical complications. Medical complications included acute cardiac event, acute pulmonary edema/failure, acute cerebrovascular event, postoperative delirium, acute renal failure, acute hepatic failure, pneumonia, sepsis, urinary tract infection, acute myocardial infarction, stroke, postoperative delirium, and Transfusion of blood. Surgical complications included periprosthetic joint infection, other postoperative infection; non-healing surgical wound; accidental perforation or laceration of a blood vessel, nerve, or organ; mechanical complication of prosthetic joint (overall analysis and separate analysis of periprosthetic fractures, prostheses loosening, and prostheses dislocation); deep vein thrombosis/pulmonary embolism (DVT/PE); injury to the peripheral nerve of the lower limb; and prosthetic revision during hospitalization. Complications of emergency and elective hospitalization in each group were compared. All complications had occurred during hospitalization and were assigned a corresponding code before discharge (Supplement Table 1).

### Statistical analysis

In this cross-sectional analysis, the analytic cohort consisted of unique and mutually exclusive patients with a procedure of THA. CCI was compared by the Kruskal-Wallis H test and pairwise compared by the Nemenyi test. Comparisons of other variables were completed with the Pearson chi-square test, and pairwise comparisons were corrected by the Bonferroni test.

Stata software, R version 3.6.2 (The R Foundation Inc) was used for data analysis. A *P* value< 0.05 with OR and 95% CI was used to determine the statistical significance of the independent variables.

## Result

Of the patients who underwent THA in the NIS database from 2005 to 2014, there were 17,200 patients with RA (2.90‰), 941 patients with AS (0.16‰), 962 patients with psoriatic arthritis (0.16‰), and 509,426 control patients with primary OA (85.94‰).

Demographics and CCI of each cohort are presented in Table 1. A cohort of RA was predominantly female and a cohort of AS was predominantly male, while the other two cohorts demonstrated approximate gender balance; All cohorts had similar age distributions. The highest percentage of patients were obese in the psoriatic arthritis group. Patients with RA underwent emergency THA more frequently. No patients who underwent prosthetic revision during the same hospitalization were observed in all groups. The highest CCI appeared in the cohort of RA, while the CCI of primary OA patients was lower than the psoriatic arthritis group but higher than ankylosing spondylitis group.

**Table 1:** Demographic characteristics of included cases

Characteristic	Rheumatoid Arthritis (%)	Psoriatic Arthritis (%)	Ankylosing Spondylitis (%)	Primary Osteoarthritis (%)	*P*-value
**Incidence rate**	2.90	0.16	0.16	85.94	–
**Female**	12,868(74.87)	490(50.94)	248(26.38)	282,952(55.68)	< 0.0001
**Age group**					< 0.0001
≤ 65	8670(50.43)	657(68.3)	661(70.24)	248,426(48.81)	
65–80	6667(38.78)	265(27.55)	232(24.65)	198,216(38.94)	
≥ 80	1856(10.8)	40(4.16)	48(5.1)	62,344(12.25)	
obesity	2263(13.16)	174(18.09)	99(10.52)	75,261(14.77)	< 0.0001
**Elective admission**	15,299(89.11)	899(93.45)	879(93.61)	479,874(94.39)	< 0.0001
**Comorbidity**	4.0734	2.986	3.606	3.174	< 0.0001

**Fig. 1:**
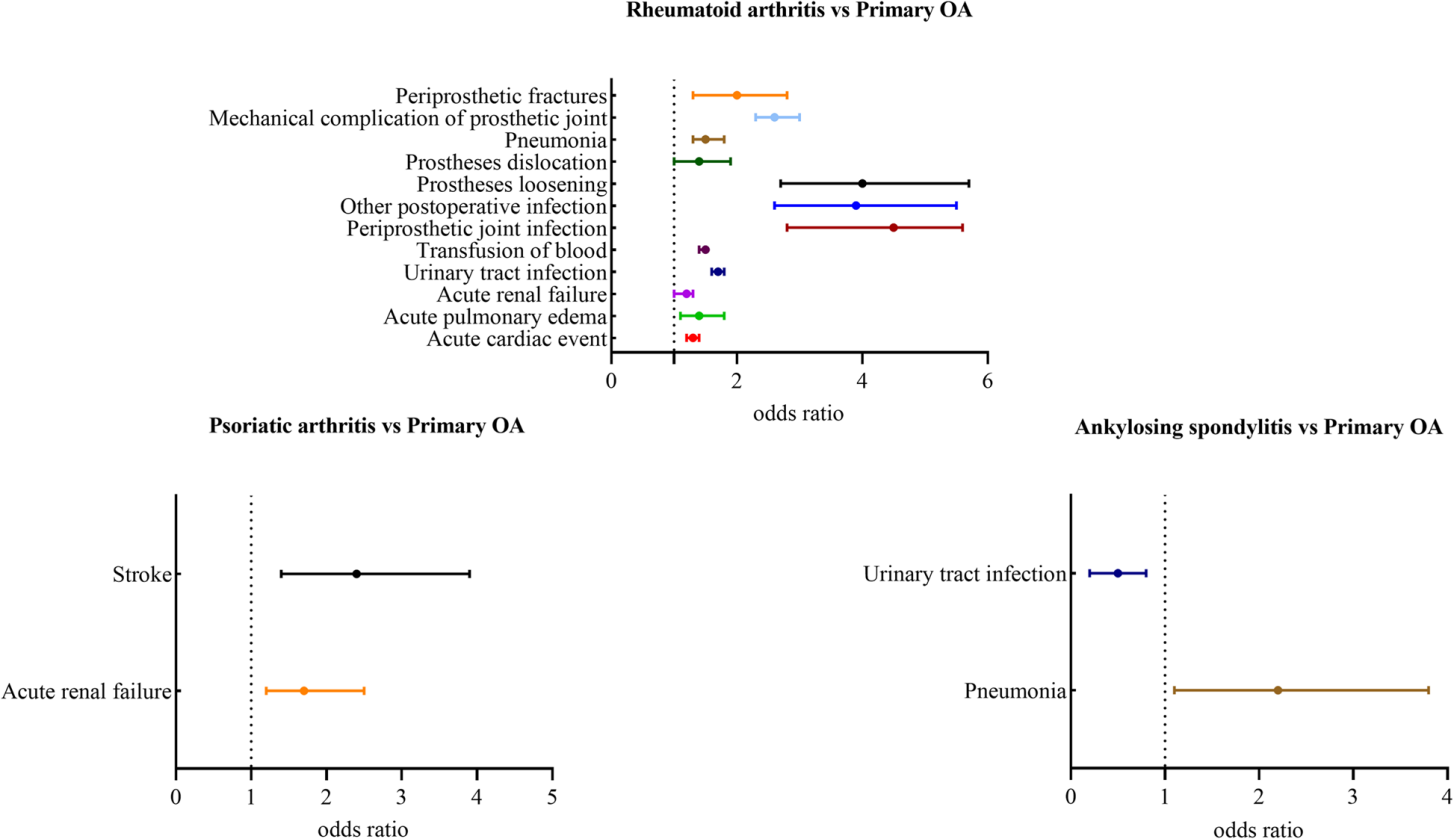
Comparison of Complication Rates in Patients with Inflammatory Arthritis and Control Patients with Osteoarthritis

In Fig. 1 or Supplement Table 2, compared to primary OA patients, postoperative medical and surgical complications were significantly more common in patients with RA than in OA patients except for acute renal failures. Specifically, the patients with RA have significantly associated with transfusion of blood (OR = 1.465 [1.416–1.516]), mechanical complications of prosthesis joint (OR = 2.582 [2.266–2.928] overall, OR = 1.951 [1.329–2.757] for periprosthetic fractures, OR = 3.990 [2.671–5.743] for mechanical loosening, OR = 1.448 [1.061–1.923] for prostheses dislocation), periprosthetic joint infection (OR = 4.544 [2.790–7.028]) and other postoperative infection (OR = 3.851 [2.58–5.539]), urinary tract infection (OR = 3.851[2.58–5.539)]), and acute cardiac event (OR = 1.325 [1.218–1.438]) acute pulmonary edema/failure (OR = 1.426 [1.098–1.816]), acute renal failure (OR = 1.175 [1.049–1.311]). Patients in the psoriatic arthritis cohort were more frequently being acute renal failure (OR = 1.745 [1.161–2.506]) or stroke (OR = 2.445 [1.401–3.923]) than in the primary OA cohort. While the rate of pneumonia (OR = 2.182 [1.128–3.757]) was higher, fewer patients with urinary tract infections (OR = 0.454 [0.243–0.766]) in AS cohort than in the OA cohort. In addition, no significant difference in the rate of postoperative DVT or PE was observed between patients with IA and primary OA patients.

**Fig. 2:**
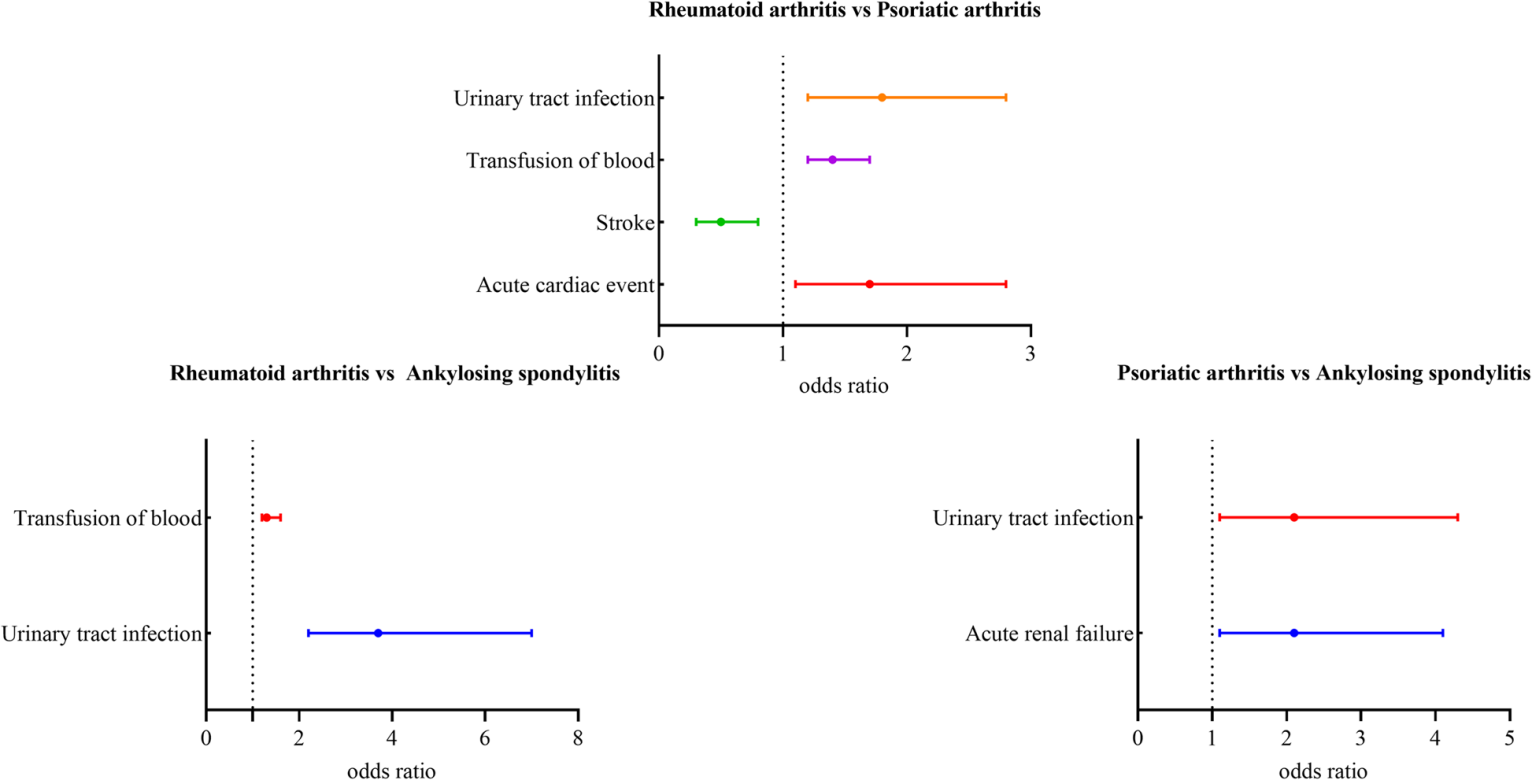
Comparison of Complication Rates Among Patients with Different types of inflammatory arthritis

In Fig. 2 or Supplement Table 3, except for several medical complications, no significant differences were found in the rates of postoperative medical and surgical complications after THA among the types of inflammatory arthritis. Compared with the RA, psoriatic arthritis was associated with lower odds of stroke (OR = 0.462 [0.279–0.826]) and higher orders of acute cardiac events (OR = 1.723 [1.13–2.793]) and transfusion of blood (OR = 1.428 [1.222–1.676]). Compared with the RA, AS was associated with higher odds of transfusion of blood (OR = 1.346 [1.153–1.579]) and urinary tract infection (OR = 3.722 [1.222–6.998]). Compared with the RA, AS was associated with higher odds of urinary tract infection (OR = 2.066 [1.053–4.281]) and acute renal failure (OR = 2.061 [1.078–4.148]).

**Fig. 3:**
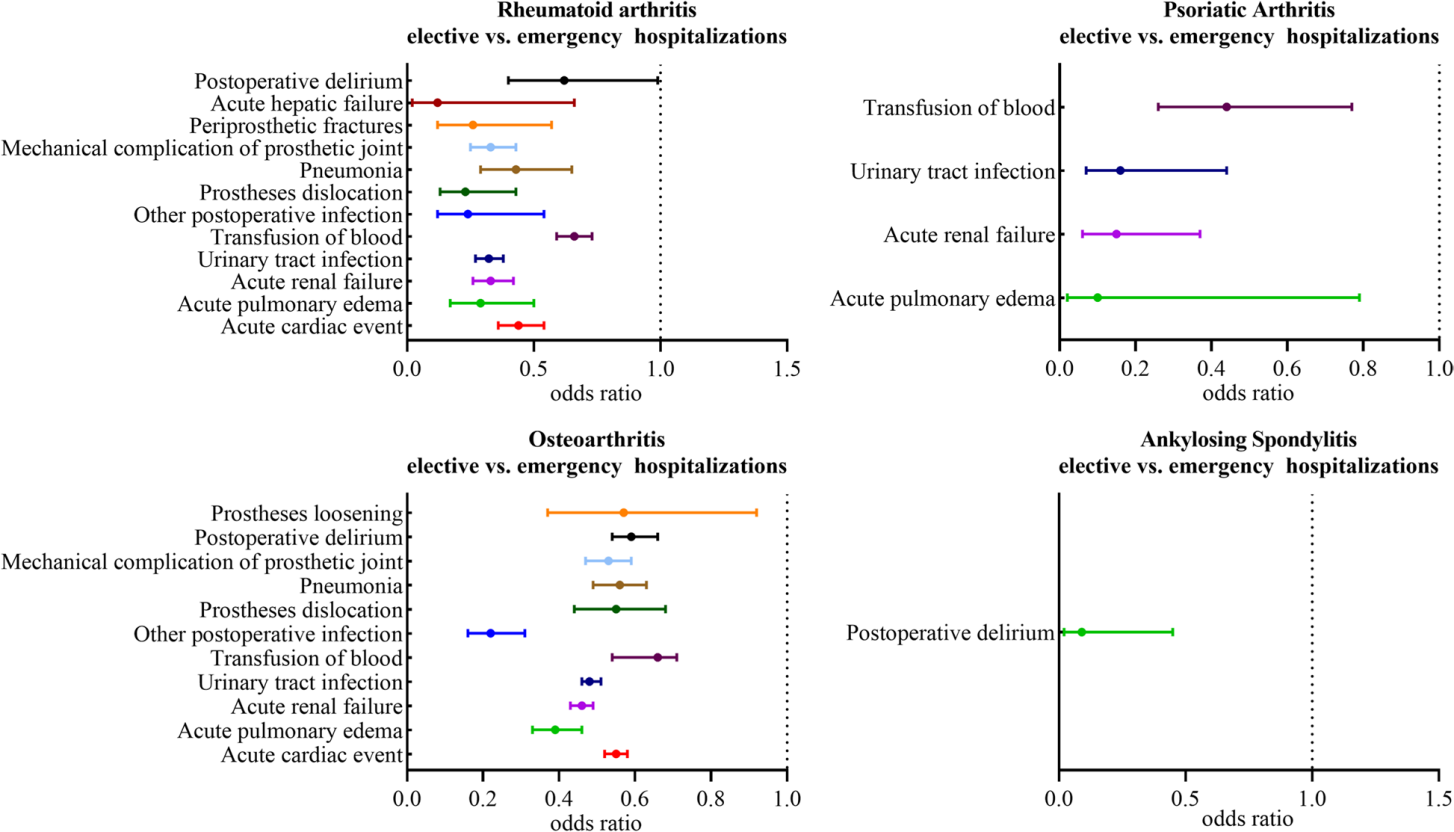
Comparison of Complication Rates in Patients in each group between emergency or elective hospitalizations

In Fig. 3 or Supplement Table 4, compared to emergency patients with RA and OA, medical complications and surgical complications were significantly less common in elective hospitalizations. The latter have significantly associated with lower incidence rate of transfusion of blood (OR = 0.655 [0.593–0.725] for RA and OR = 0.707 [0.688–0.726] for OA), other postoperative infection (OR = 0.243 [0.116–0.543] for RA and OR = 0.224 [0.164–00.311] for OA), mechanical complications of prosthesis joint (OR = 0.325 [0.247–0.432] for RA and OR = 0.525 [0.466–0.592] for OA overall, OR = 0.228 [0.126–0.428] for RA and OR = 0.548 [0.444–0.684] for OA of prostheses dislocation, OR = 0.256 [0.123–0.567] for periprosthetic fractures of RA, OR = 0.572 [0.374–0.923] for periprosthetic fractures of OA, no significant difference for periprosthetic fractures of OA or mechanical loosening of RA), delirium (OR = 0.619 [0.403–0.994] for RA and OR = 0.595 [0.537–0.661] for OA), acute cardiac event (OR = 0.438 [0.359–0.537] for RA and OR = 0.546 [0.516–0.579] for OA), acute pulmonary edema/failure (OR = 0.287 [0.170–0.503] for RA and OR = 0.388 [0.332–0.456] for OA), acute renal failure (OR = 0.326 [0.255–0.421] for RA and OR = 0.460 [0.430–0.494] for OA), pneumonia (OR = 0.429 [0.291–0.652] for RA and OR = 0.556 [0.491–0.633] for OA), urinary tract infection (OR = 0.323 [0.274–0.383] for RA and OR = 0.481 [0.455–0.508] for OA). Besides, different from RA, sepsis (OR = 0.297 [0.124–0.881]) was less common in selective admission patients with OA than in emergency patients, while selective RA patients were observed with a lower rate of acute hepatic failure (OR = 0.122 [0.023–0.660]). Moreover, with elective hospitalizations, the rate of acute renal failure (OR = 0.148 [0.064–0.374]), urinary tract infection (OR = 0.163 [0.068–0.435]), acute pulmonary edema/failure (OR = 0.102 [0.017–0.786], and transfusion of blood (OR = 0.441 [0.260–0.766]) was lower in psoriatic arthritis cohort than with emergency hospitalizations. Finally, for patients with ankylosing spondylitis, elective hospitalizations showed a lower incidence rate of postoperative delirium (OR = 0.087 [0.019–0.45]) only.

## Discussion

The THA procedure is an effective surgical strategy to manage pain and restore function and mobility in patients with end-stage arthritis of the hip [15]. Patients with IAs, including RA, psoriatic arthritis, and AS, have multisystem disease manifestations that accompany hip arthritis and are at higher risk of complications after lower extremity arthroplasty [3]. Primary THA in patients with IAs has not been well defined, and previous studies have been limited to small institutional case series. The current study provides a comprehensive comparison of complications after THA in patients with IAs using a large national database.

In this study, except for RA, there was not many significant acute postoperative complications difference between psoriatic arthritis and primary OA or between AS and primary OA. There were also only several postoperative medical complications differences between the three different IAs in this study, which is not consistent with the study’s hypotheses.

For the patients undergoing THA, patients with primary OA account for a large proportion compared with IAs. and RA accounts for a large proportion of IAs undergoing total hip replacement. RA and AS show two extremes in sex distribution, the former is mainly female and the latter is male. Psoriatic arthritis and AS are also more inclined in younger non-obese patients with fewer comorbidities. This can also explain the higher rate of elective admission and the fewer postoperative complications of elective patients. Psoriasis arthritis was reported strongly associated with obesity, which increases the risk of surgical site infection and deep vein thrombosis [16]. However, in this study, the rate of obesity in patients with Psoriatic arthritis is just nearly 18%, which could also account for the reason why there is no difference in the periprosthetic joint infection and other postoperative infection and DVT/PE between psoriatic arthritis and OA.

When comparing IAs with primary OA, like in other studies, postoperative medical and surgical complications were significantly more common in patients with rheumatoid arthritis [17]. Both for OA and the other two comparisons, RA showed a strong association with blood transfusion, especially in emergency patients, similar observations have previously been published for total knee and shoulder arthroplasty [18, 19]. The increased requirement for blood product substitution is believed to partly relate to iron-deficiency anemia caused by chronic non-steroidal analgesic use, anemia of chronic disease and bone marrow depression due to medications, all of which are common in RA patients. In addition, medications also led to higher postoperative infection rates in RA patients, which were higher in emergency patients, requiring more comprehensive postoperative infection prevention regimens. Furthermore, in this study, compared with OA, the mechanical complications of prosthetic joint of RA are significantly increased, including periprosthetic fractures, prostheses loosening, and prostheses dislocation, which was worse in emergency patients. It might also be related to the negative effect of long-term corticosteroid treatment on bone and bone marrow and the age of emergency RA patients is generally older and have more fragile muscle ligaments and bone mass.

For psoriatic arthritis, it should be noted that the American Academy of Orthopaedic Surgeons’ clinical practice guidelines cite immunosuppression (including psoriatic arthropathy) as a risk factor for periprosthetic infection of the knee (but not the hip), as supported by a systematic review of the available evidence, whereas skin psoriasis has been posited as a risk factor by consensus of the American Academy of Orthopaedic Surgeons work group [16]. Yet, in this study, there is no difference in wound healing between patients with psoriatic arthritis and OA or other two types of IAs, which more support the opinion of the former. Psoriasis has also been identified as an independent risk factor for myocardial infarction which may account for the result in this study that psoriatic arthritis is highly associated with stroke. Patients in the psoriatic arthritis cohort were more frequently being acute renal failure or stroke than in the OA cohort. Besides, emergency patients with psoriatic arthritis are more prone to pulmonary and urinary complications. It means that in the perioperative period, compared with primary OA, more attention should be paid to the protection of renal and pulmonary function and shock in patients with psoriatic arthritis, such as increasing the preoperative perfusion volume and promoting pulmonary function exercise.

Interestingly, for AS, whether compared with OA, RA, or psoriatic arthritis, it is the protective factor against urinary tract infection, which is not consistent with other studies [20, 21]. The reason for this phenomenon may be the result of clinicians understanding of the correlation between AS and urinary tract infection and preventing it in advance.

Our study has several limitations. This study roughly revealed the different impacts of IAs and primary OA on hip replacement. First of all, the relatively small size of IAs patients provided limited statistical power. In addition, the period of data we could choose to observe, between 2005 and 2014, was limited by the ICD-9-CM system, which was updated to ICD-10-CM in NIS from 2015 to 2020 availability. Due to the involvement of psoriatic arthritis and ankylosing spondylitis, which are relatively rare, we choose this period to increase the sample size which was 10 years from 2005 and 2014 rather than 5 years from 2015 to 2020. Moreover, the severity of the different IAs and the approaches to operations had been limited by the database using the ICD-9-CM code. Finally, because the re-admission indicators and long-term complications were not included in the NIS database, result in statistical deviations in the revision rate.

## Conclusion

Except for patients with RA, the differences in perioperative complications were small between patients with IA and primary OA and between patients with different types of IA. What is more, specific medical complications including transfusion of blood for RA and stroke for psoriatic arthritis need to be paid more attention to in clinical practice. Except for patients with RA, no significant differences were found in perioperative surgical complications between different types of IAs and primary OA. No significant differences were found in perioperative surgical complications among the types of inflammatory arthritis.

## Supplementary Information

**Additional file 1: Supplement Table 1. Supplement Table 2.** Comparison of Complication Rates in Patients with Inflammatory Arthritis and Control Patients with Osteoarthritis. **Supplement Table 3.** Comparison of Complication Rates Among Patients with Different types of inflammatory arthritis. **Supplement Table 4.** Comparison of Complication Rates in Patients in each group between emergency or elective hospitalizations.

## Data Availability

These data are easily available from the Agency for Healthcare Research and Quality (AHRQ’s) “Healthcare Cost and Utilization Project (HCUP)” and can be obtained after completing an on-line Data Use Agreement training session and signing a Data Use Agreement. The contact information for requesting the data is as follows: HCUP Central Distributor. Phone: (866) 556–4287 (toll-free). Fax: (866) 792–5313. E-mail: HCUPDistributor@ahrq.gov.

## Abbreviations

THA: Total hip arthroplasty
OR: Odds ratio
NIS: Nationwide Inpatient Sample
DVT/PE: Deep vein thrombosis/pulmonary embolism
IAs: Inflammatory arthropathies
RA: Rheumatoid arthritis
AS: Ankylosing spondylitis
OA: Osteoarthritis
