# Supplementary material for: Complications of primary total hip arthroplasty among patients with rheumatoid arthritis, psoriatic arthritis, ankylosing spondylitis, and primary osteoarthritis
Source: BMC Musculoskelet Disord. 2022 Oct 19;23:924. doi: 10.1186/s12891-022-05891-9 (PMC9580196; doi:10.1186/s12891-022-05891-9)
Supplement: Supplementary file 1 — Additional file 1: Supplement Table 1. Supplement Table 2. Comparison of Complication Rates in Patients with Inflammatory Arthritis and Control Patients with Osteoarthritis. Supplement Table 3. Comparison of Complication Rates Among Patients with Different types of inflammatory arthritis. Supplement Table 4. Comparison of Complication Rates in Patients in each group between emergency or elective hospitalizations. [file 12891_2022_5891_MOESM1_ESM.docx]

| Supplement Table 1 | |
| --- | --- |
| Variable | ICD-9 Code |
| **Acute complications** |  |
| **Surgical complications** |  |
| Shock | 998.0 |
| Hemorrhage, hematoma, or seroma | 998.1, 998.11, 998.12, 998.13 |
| Accidental perforation or laceration of blood vessel, nerve, or organ | 998.2 |
| Wound dehiscence | 998.3, 998.30, 998.31, 998.32, 998.33 |
| Periprosthetic joint infection | 996.66, 996.67 |
| Other postoperative infection | 711，7110，71100，71105，7116，71160，7119，71190，71195，730，73000，73005，7301，73010，73015，7302，73025，7309，73090，73095 |
| Fracture of neck, shaft, or unspecified - femur | 820，8200，82001，82003，82009，8201，82010，82011，82012，82013，82019，8202，82020，82021，82022，8203，82030，8208，8209，8210，82100，82101，8211，82110，82111 |
| Non-healing surgical wound | 998.83 |
| Other unspecified procedural complications | 998.8, 998.81, 998.89, 998.9 |
| DVT/PE | 41511，41519，45340，45341，45342 |
| Mechanical complication of prosthetic joint | 99640-99647，99649 |
| Prostheses dislocation | 996.42 |
| Periprosthetic fractures | 996.44 |
| Prostheses loosening | 996.41, 996.43, 996.45 |
| Injury to peripheral nerve of lower limb | 956, 956.0, 956.1, 956.2, 956.3, 956.4, 956.5, 956.8, 956.9 |
| **Medical complications** |  |
| Acute cardiac event | 410.0-410.9, 411.1, 411.8, 415.0, 420.0, 420.9, 421.0, 421.1, 421.9, 422.0, 422.9, 427.0-427.5, 428.0-428.9 |
| Acute pulmonary edema/failure | 518.4, 518.81, 518.82, 518.84 |
| Acute cerebrovascular event | 997.00, 997.01, 997.02, 997.09 |
| Acute renal failure | 584.5-584.9 |
| Acute hepatic failure | 570 |
| Pneumonia | 480,480.0, 480.1, 480.2, 480.3, 480.8, 480.9, 481, 482, 482.0, 482.1, 482.3, 482.30, 482.31, 482.32, 482.39, 482.40, 482.41, 482.42, 482.49, 482.8, 482.81, 482,82, 482.83, 482.84, 482.89, 482.9, 483, 483.1, 483.8, 484, 484.1, 484.3, 484.5, 484.6, 484.7, 484.8, 485, 487.0, V12.61, 507.0, 514, 518.4, 518.5, 516, 516.8, 997.31 |
| Sepsis | 995.9, 038.0-038.4, 999.3 |
| Urinary tract infection | 599.0, 996.64, 996.31, V13.02 |
| Acute myocardial infarction | 410,410.0,410.1,410.2,410.3,410.4,410.5,410.6,410.7,410.8,410.9,997.1 |
| Postoperative delirium | 293,293.0,293.1,293.8,293.9,293.81,293.82,293.83,293.84,293.89,292.81,780.97 |
| Sepsis | 995.91/995.92 |
| Transfusion of blood | 99.00,99.02,99.03,99.04,99.05,99.07,99.08 |
| Stroke | 430,431,432,432.0,432.1,432.9,433,433.0,433.00,433.01,433.1,433.10,433.11,433.2,433.20,433.21,433.3,433.30,433.31,433.30,433.31,433.8,433.80,433.81,433.9,433.90,433.91,434,434.0,434.00,434.01,434.1,434.10,434.11,434.9,434.90,434.91,435,435.0,435.1,435.2,435.3,435.8,435.9,997.02 |

| Supplement Table 2: Comparison of Complication Rates in Patients with Inflammatory Arthritis and Control Patients with Osteoarthritis | | | | | | |
| --- | --- | --- | --- | --- | --- | --- |
| Complication | Rheumatoid Arthritis VS Osteoarthritis | | Psoriatic Arthritis VS Osteoarthritis | | Ankylosing Spondylitis VS Osteoarthritis | |
|  | Odds Ratio (95% CI) | P Value | Odds Ratio (95% CI) | P Value | Odds Ratio (95% CI) | P Value |
| **Medical complications** |  |  |  |  |  |  |
| Acute cardiac event | 1.325(1.218,1.438) | **<0.001** | 0.769(0.477,1.163) | 0.245 | 0.867(0.551,1.289) | 0.508 |
| Acute pulmonary edema/failure | 1.426(1.098,1.816) | **0.006** | 1.994(0.713,4.309) | 0.124 | 2.039(0.729,4.406) | 0.113 |
| Acute cerebrovascular event | 1.124(0.712,1.676) | 0.592 | 0.913(0.052,4.035) | 0.928 | 1.869(0.31,5.8) | 0.378 |
| Acute renal failure | 1.175(1.049,1.311) | **0.005** | 1.745(1.161,2.506) | **0.004** | 0.847(0.464,1.401) | 0.552 |
| Acute hepatic failure | 1.851(0.722,3.873) | 0.143 | 5.521(0.313,24.768) | 0.089 | 0(0,0.013) | 0.95 |
| Pneumonia | 1.525(1.281,1.799) | **<0.0001** | 1.352(0.5792.626) | 0.427 | 2.182(1.128,3.757) | **0.01** |
| Sepsis | 1.975(0.319,6.532) | 0.352 | 0.001(0,0.088) | 0.941 | 0.001(0,0.09) | 0.941 |
| Urinary tract infection | 1.691(1.571,1.819) | **<0.0001** | 0.939(0.614,1.365) | 0.755 | 0.454(0.243,0.766) | **0.007** |
| Acute myocardial infarction | 0.989(0.816,1.185) | 0.904 | 0.922(0.365,1.875) | 0.843 | 1.737(0.899,2.991) | 0.069 |
| Pulmonary embolism | 1.113(0.766,1.556) | 0.554 | 0(0,0) | 0.934 | 0.635(0.036,2.804) | 0.65 |
| Stroke | 1.13(0.94,1.345) | 0.181 | 2.445(1.401,3.923) | **0.001** | 0.991(0.393,2.016) | 0.982 |
| Postoperative delirium | 0.947(0.796,1.116) | 0.525 | 0.967(0.441,1.808) | 0.926 | 0.865(0.371,1.679) | 0.702 |
| Transfusion of blood | 1.465(1.416,1.516) | **<0.0001** | 1.026(0.877,1.195) | 0.744 | 1.089(0.931,1.267) | 0.279 |
| **Surgical complications** |  |  |  |  |  |  |
| Periprosthetic joint infection | 3.851(2.58,5.539) | **<0.0001** | 2.294(0.13,10.183) | 0.408 | 2.345(0.133,10.411) | 0.395 |
| Other postoperative infection | 3.851(2.58,5.539) | **<0.0001** | 2.294(0.13,10.183) | 0.408 | 2.345(0.133,10.411) | 0.395 |
| Non-healing surgical wound | 1.185(0.066,5.585) | 0.868 | 0.001(0,0.206) | 0.946 | 0.001(0,0.21) | 0.946 |
| Accidental perforation or laceration of blood vessel, nerve, or organ | 1.706(0.874,2.982) | 0.085 | 2.774(0.158,12.336) | 0.309 | 0(0,0) | 0.93 |
| Mechanical complication of prosthetic joint | 2.582(2.266,2.928) | **<0.0001** | 1.601(0.767,2.901) | 0.16 | 1.454(0.662,2.719) | 0.293 |
| Prostheses dislocation | 1.448(1.061, 1.923) | **0.0144** | 1.125(0.187, 3.486) | 0.868 | 1.726(0.428, 4.498) | 0.346 |
| Periprosthetic fractures | 1.951(1.329,2.757) | **0.0003** | 1.124(0.064,4.973) | 0.907 | 2.302(0.382,7.150) | 0.240 |
| Prostheses loosening | 3.990(2.671,5.743) | **<0.0001** | 2.376(0.135,10.552) | 0.388 | 2.429(0.138,10.788) | 0.376 |
| DVT/PE | 1.188(0.918,1.51) | 0.173 | 0.98(0.243,2.55) | 0.972 | 1.337(0.414,3.12) | 0.562 |
| Injury to the peripheral nerve of lower limb | 0.8(0.316,1.645) | 0.591 | 0(0,0) | 0.924 | 0(0,0) | 0.924 |

| Supplement Table 3: Comparison of Complication Rates Among Patients with Different types of inflammatory arthritis | | | | | | |
| --- | --- | --- | --- | --- | --- | --- |
| Complication | Rheumatoid Arthritis VS Psoriatic Arthritis | | Rheumatoid Arthritis VS Ankylosing Spondylitis | | Psoriatic Arthritis VS Ankylosing Spondylitis | |
|  | Odds Ratio (95% CI) | P Value | Odds Ratio (95% CI) | P Value | Odds Ratio (95% CI) | P Value |
| **Medical complications** |  |  |  |  |  |  |
| Acute cardiac event | 1.723(1.13,2.793) | **0.018** | 1.528(1.019,2.421) | 0.053 | 0.887(0.477,1.639) | 0.701 |
| Acute pulmonary edema/failure | 0.715(0.317,2.047) | 0.471 | 0.699(0.31,2.002) | 0.442 | 0.978(0.271,3.527) | 0.972 |
| Acute cerebrovascular event | 1.231(0.258,22.055) | 0.839 | 0.601(0.177,3.757) | 0.491 | 0.489(0.023,5.109) | 0.559 |
| Acute renal failure | 0.673(0.461,1.025) | 0.051 | 1.388(0.829,2.554) | 0.25 | 2.061(1.078,4.148) | **0.034** |
| Acute hepatic failure | 0.335(0.057,6.337) | 0.312 | 15688.612(0.047,NA) | 0.942 | 280697.185(0,NA) | 0.969 |
| Pneumonia | 1.128(0.568,2.667) | 0.757 | 0.699(0.395,1.375) | 0.255 | 0.62(0.227,1.58) | 0.324 |
| Sepsis | 5898.891(0,NA) | 0.95 | 5770.121(0,NA) | 0.95 | - | - |
| Urinary tract infection | 1.802(1.231,2.769) | **0.004** | 3.722(2.198,6.998) | **<0.0001** | 2.066(1.053,4.281) | **0.041** |
| Acute myocardial infarction | 1.072(0.514,2.746) | 0.868 | 0.569(0.32,1.124) | 0.076 | 0.531(0.182,1.4) | 0.214 |
| Pulmonary embolism | 118691.119(60.335+25) | 0.94 | 1.752(0.377,31.193) | 0.58 | 0(NA,1.89139943637) | 0.969 |
| Stroke | 0.462(0.279,0.826) | **0.005** | 1.141(0.548,2.918) | 0.753 | 2.468(0.999,6.947) | 0.063 |
| Postoperative delirium | 0.979(0.511,2.18) | 0.953 | 1.095(0.551,2.59) | 0.815 | 1.119(0.4,3.203) | 0.829 |
| Transfusion of blood | 1.428(1.222,1.676) | **<0.0001** | 1.346(1.153,1.579) | **<0.0001** | 0.943(0.758,1.172) | 0.595 |
| **Surgical complications** |  |  |  |  |  |  |
| Periprosthetic joint infection | 2839527.000(0,NA) | 0.987 | 2839527.000(0,NA) | 0.988 | - | - |
| Other postoperative infection | 1.679(0.36,29.921) | 0.61 | 1.642(0.352,29.268) | 0.625 | 0.978(0.039,24.76) | 0.988 |
| Non-healing surgical wound | 5898.548(0,NA) | 0.965 | 5769.785(0,NA) | 0.965 | - | - |
| Accidental perforation or laceration of blood vessel, nerve, or organ | 0.615(0.119,11.248) | 0.642 | 42658.623(0.272,NA) | 0.947 | 280697.185(0,NA) | 0.969 |
| Mechanical complication of prosthetic joint | 1.613(0.879,3.395) | 0.161 | 1.776(0.938,3.929) | 0.111 | 1.101(0.419,2.945) | 0.843 |
| Prostheses dislocation | 1.287(0.398, 7.890) | 0.727 | 0.838(0.306,3.458) | 0.768 | 0.651(0.086,3.940) | 0.639 |
| Periprosthetic fractures | 1.735(0.372,30.905) | 0.588 | 0.848(0.256,5.241) | 0.821 | 0.488(0.023,5.109) | 0.559 |
| Prostheses loosening | 1.679(0.360,29.922) | 0.610 | 1.642(0.352,29.268) | 0.626 | 0.978(0.039,24.760) | 0.988 |
| DVT/PE | 1.213(0.45,4.967) | 0.744 | 0.889(0.366,2.928) | 0.819 | 0.733(0.144,3.333) | 0.685 |
| Injury to peripheral nerve of lower limb | 16038.73(0.048,NA) | 0.942 | 15688.612(0.047,NA) | 0.942 | - | - |

| Supplement Table 4: Comparison of Complication Rates in Patients in each group between emergency or elective hospitalizations. | | | | | | | | |
| --- | --- | --- | --- | --- | --- | --- | --- | --- |
| Complication | Rheumatoid Arthritis (elective VS emergency hospitalizations) | | Psoriatic Arthritis (elective VS emergency hospitalizations) | | Ankylosing Spondylitis (elective VS emergency hospitalizations) | | Osteoarthritis  (elective VS emergency hospitalizations) | |
|  | Odds Ratio (95% CI) | P Value | Odds Ratio (95% CI) | P Value | Odds Ratio (95% CI) | P Value | Odds Ratio (95% CI) | P Value |
| **Medical complications** |  |  |  |  |  |  |  |  |
| Acute cardiac event | 0.438(0.359,0.537) | **<0.0001** | 0.385(0.125,1.684) | 0.140 | 0.420(0.138,1.824) | 0.170 | 0.546(0.516,0.579) | **<0.0001** |
| Acute pulmonary edema/failure | 0.287(0.170,0.503) | **<0.0001** | 0.102(0.017,0.786) | **0.013** | 4888646.593(0,NA) | 0.995 | 0.388(0.332,0.456) | **<0.0001** |
| Acute cerebrovascular event | 0.415( 0.164,1.263) | 0.0840 | 2586712.812(0,NA) | 1.000 | 1948769.497(0,NA) | 0.995 | 0.799(0.588,1.12) | 0.170 |
| Acute renal failure | 0.326(0.255,0.421) | **<0.0001** | 0.148(0.064,0.374) | **<0.0001** | 0.368(0.096,2.411) | 0.200 | 0.460(0.430,0.494) | **<0.0001** |
| Acute hepatic failure | 0.122(0.023,0.660) | **0.0100** | 0(NA,Inf) | 1.000 | - | - | 0.511(0.279,1.049) | -- |
| Pneumonia | 0.429(0.291,0.652) | **<0.0001** | 0.171(0.036,1.208) | - | 0.679(0.127,12.565) | 0.710 | 0.556(0.491,0.633) | **<0.0001** |
| Sepsis | 0.122( 0.005,3.089) | 0.137 | - | - | - | - | 0.297(0.124,0.881) | **0.013** |
| Urinary tract infection | 0.323(0.274,0.383) | **<0.0001** | 0.163(0.068,0.435) | **<0.0001** | 0.748(0.142,13.783) | 0.780 | 0.481(0.455,0.508) | **<0.0001** |
| Postoperative delirium | 0.619(0.403,0.994) | **0.036** | 0.487(0.085,9.168) | 0.500 | 0.087(0.019,0.45) | **0.002** | 0.595(0.537,0.661) | **<0.0001** |
| Transfusion of blood | 0.655(0.593,0.725) | **<0.0001** | 0.441(0.260,0.766) | **0.003** | 0.943(0.521,1.813) | 0.850 | 0.707(0.688,0.726) | **<0.0001** |
| **Surgical complications** |  |  |  |  |  |  |  |  |
| Periprosthetic joint infection | 0.243(0.116,0.543) | **0.0003** | 2586712.845(0,NA) | 1.000 | 2645635.678(0,NA) | 1.000 | 0.224(0.164,0.311) | **<0.0001** |
| Other postoperative infection | 0.390(0.153,1.194) | 0.0666 | #### | ### | #### | ### | 0.567(0.333,1.055) | 0.0516 |
| Non-healing surgical wound | 0.000(NA,1.581*10^267)^ | 0.990 | 1.000(0,NA) | 1.000 | 1.000(0,NA) | 1.000 | 0.684(0.202,4.261) | 0.606 |
| Accidental perforation or laceration of blood vessel, nerve, or organ | 0.488(0.122,3.237) | 0.360 | 2586712.836(0,NA) | 1.000 | 1.000(0,NA) | 1.000 | 1.345(0.709,2.979) | 0.412 |
| Mechanical complication of prosthetic joint | 0.325(0.247,0.432) | **<0.0001** | 0.082(0.021,0.341) | **0.0003** | 0.199(0.045,1.381) | 0.051 | 0.525(0.466,0.592) | **<0.0001** |
| Prostheses dislocation | 0.228(0.126,0.428) | **<0.0001** | 1905318.674(0,NA) | 0.990 | 0.135(0.013,2.92) | 0.100 | 0.548(0.444,0.684) | **<0.0001** |
| Periprosthetic fractures | 0.256(0.123,0.567) | **0.0004** | 2586712.842(0,NA) | 1.000 | 0.067(0.003,1.713) | 0.057 | 0.940(0.655,1.41) | 0.753 |
| Prostheses loosening | 3.425(0.731,61.087) | 0.230 | 2586712.829(0,NA) | 1.000 | 2645635.699(0,NA) | 1.000 | 0.572(0.374,0.923) | **0.0148** |
| Injury to peripheral nerve of lower limb | 2477294.312(0.000,NA) | 0.990 | - | - | -- | - | 0.717(0.451,1.221) | 0.187 |
